# Supplementary material for: Boosting Lithium Storage of a Metal-Organic Framework via Zinc Doping
Source: Materials (Basel). 2022 Jun 13;15(12):4186. doi: 10.3390/ma15124186 (PMC9227496; doi:10.3390/ma15124186)
Supplement: Supplementary file 1 [file materials-15-04186-s001.zip › materials-1731917-supplementary.pdf]

Supporting information of Boosting lithium storage of a metal-organic framework via zinc doping

Wenshan Gou[1], Zhao Xu[1], Xueyu Lin[2*], Yifei Sun[1], Xuguang Han[1], Mengmeng Liu[1], Yan Zhang[1*]

1 Institute of Advanced Cross-field Science, College of Life Sciences, Qingdao University, Qingdao 200671, P. R. China

2 Beijing National Laboratory for Molecular Sciences and State Key Laboratory of Rare Earth Materials Chemistry and Applications, College of Chemistry and Molecular Engineering, Peking University, Beijing 100871, P.R. China

Corresponding authors: yzhang_iacs@qdu.edu.cn; 1801110301@pku.edu.cn

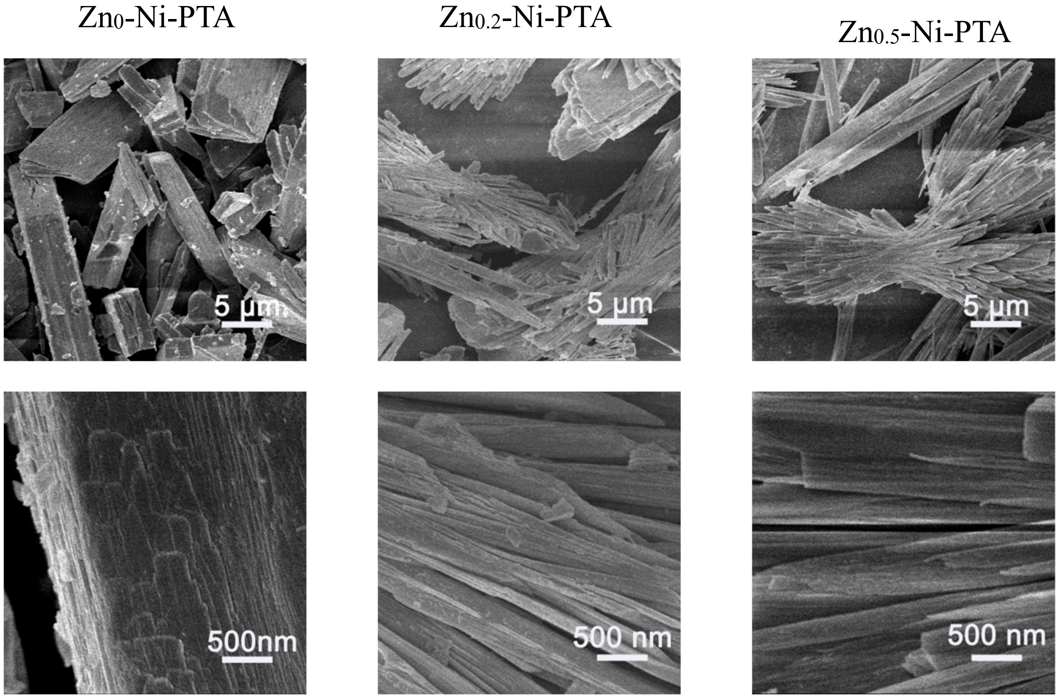

Figure S1. SEM of the samples.

Table S1. Induced coupled plasma atomic emission spectroscopy (ICP-AES) results of the Zn-doped Ni-MOF.

	Ni^{2+} (mmol)	Zn^{2+} (mmol)	Atom fraction %($Zn^{2+}/(Ni^{2+}+Zn^{2+})$)
Zn_0-Ni-PTA	1.50	0	-
$Zn_{0.1}$-Ni-PTA	1.35	0.15	10.97%
$Zn_{0.2}$-Ni-PTA	1.20	0.30	22.06%
$Zn_{0.3}$-Ni-PTA	1.05	0.45	31.18%
$Zn_{0.4}$-Ni-PTA	0.90	0.60	41.97%
$Zn_{0.5}$-Ni-PTA	0.75	0.75	50.83%

Table S2. The surface area (BET) of the Zn_x-Ni-MOF.

	Zn_0-Ni-PTA	$Zn_{0.2}$-Ni-PTA	$Zn_{0.5}$-Ni-PTA
S_{BET} (m^2 g^{-1})	2.8	3.3	3.6

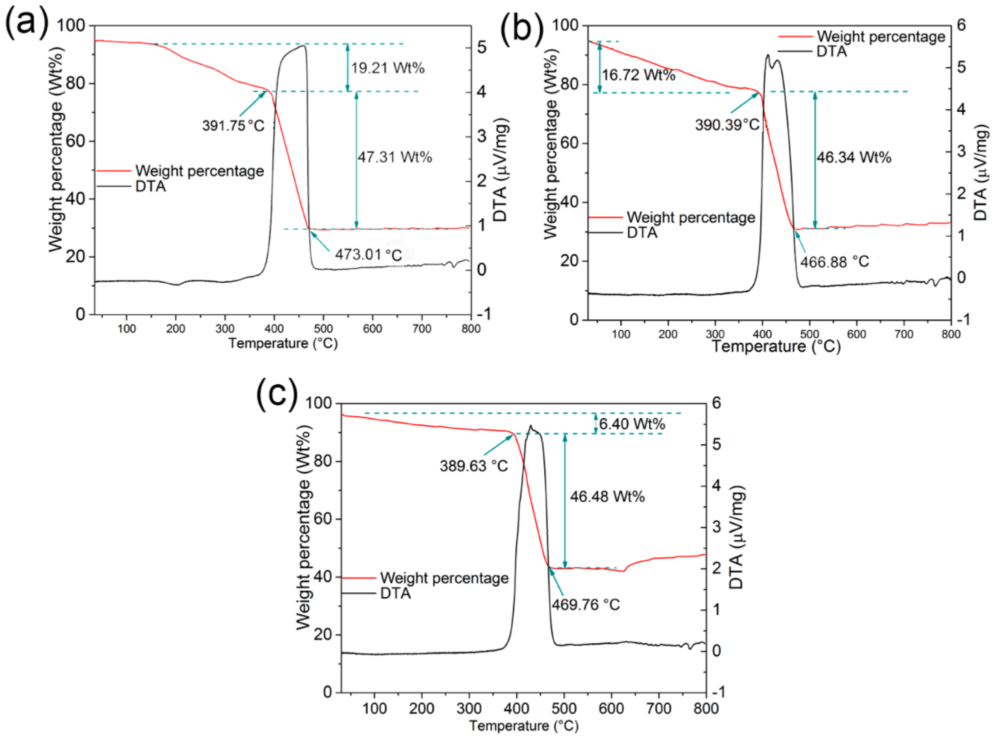

Figure S2. TG-DTA of the (a) 0% Zn-doped Ni-MOF, (b) 20% Zn-doped Ni-MOF, (c) 50% Zn-doped Ni-MOF.

(a) 0% Zn-doped Ni-MOF

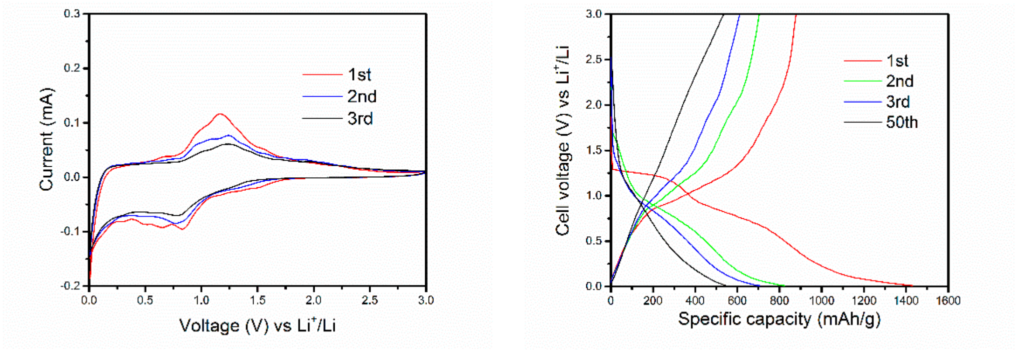

(b) 50% Zn-doped Ni-MOF

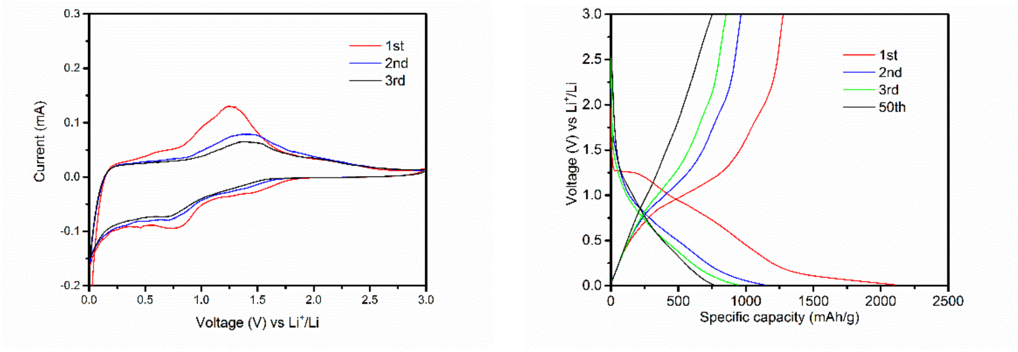

Figure S3. CV (left) and charge/discharge curves (right) of the samples.

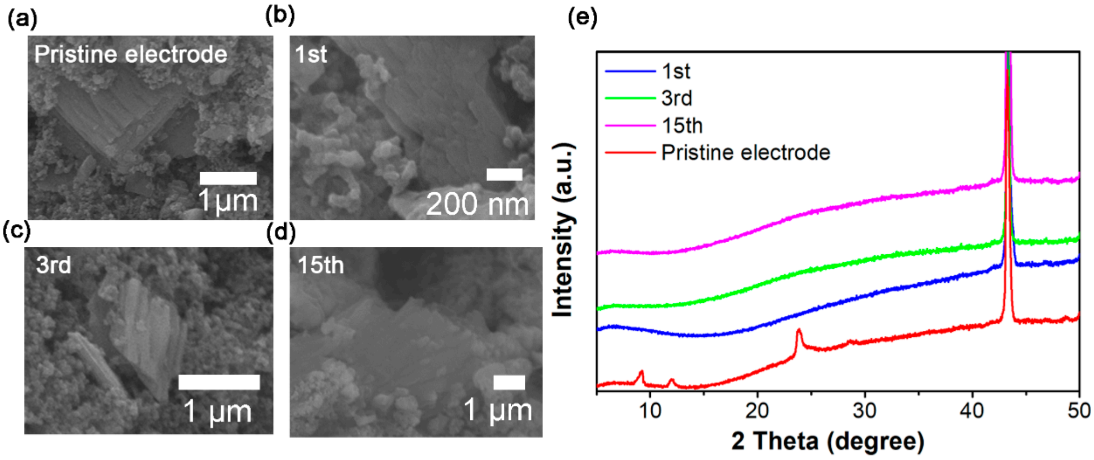

Figure S4. (**a**,**b**,**c**,**d**) SEM micrograph of electrode, (**e**) XRD patterns of the electrodes.

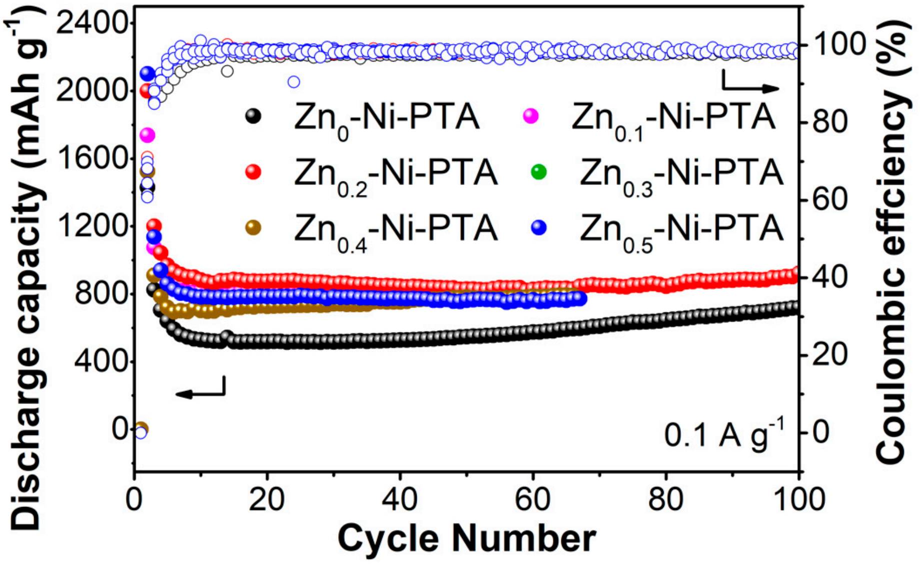

Figure S5.The cycle performance of different amounts of Zn-doped Ni-PTA.

Table S3. Summary of metal–organic frameworks reported as anode materials in rechargeable lithium-ions batteries.

**MOF Anode**	**Voltage (V vs. Li/Li$^+$)**	**Current Density (mA g^{-1})**	**Capacity (mAh g^{-1})**	**Cycle**	**Reference**
MOF-177 Zn_4O(1,3,5-benzenetribenzoate)$_2$	0.05-1.6 V	50	20	50	[1]
Cu-BDC	0.01-2.5 V	48	161	50	[2]
UIO-66 (Zr6O4(OH)4)4(BDC)	0.02-3.0 V	38.8	85	100	[3]
Ni-NTC	0.01-3.0 V	100	238	80	[4]
Zn(IM)1.5(abIM)0.5	0.01-3.0 V	100	190	200	[5]
MIL-101(Cr)	0.01-3.25 V	50	24	40	[6]
Pb-MOF	0.01-3 V	100	489	500	[7]
MIL-101(Cr)/graphene oxid	0.01-3.25 V	50	40	40	[6]
Zn1.5Co1.5(HCOO)6	0.005-3.0 V	60	510	60	[8]
Mn-LCP Mn(tfbdc)(4,40-bpy)(H2O)2	0.01-2.5 V	50	390	50	[9]
Co-Zn-ZIF-67	0.01-3.0 V	100	605	100	[10]
MOF-74 (Fe, Co, Ni))	0.1-3.0 V	1000	456	500	[11]
Tb-MOF	0.01-3.0 V	100	395	100	[12]
Zn-Ni-PTA Zn0.6Ni2.4(OH)2(C8H4O4)2·(H2O)4	0.0–3.0 V	100	921	100	This work

Reference

[1]. X. X. Li, F. Y. Cheng, S. N. Zhang, J. Chen, Shape-controlled synthesis and lithium-storage study of metal-organic frameworks Zn_4O(1,3,5-benzenetribenzoate)(2), Journal of Power Sources 160, (2006) 542-547. http://doi.org/10.1016/j.jpowsour.2006.01.015

[2]. R. Senthil Kumar, C. Nithya, S. Gopukumar, M. Anbu Kulandainathan, Diamondoid-Structured Cu-Dicarboxylate-based Metal-Organic Frameworks as High-Capacity Anodes for Lithium-Ion Storage, Energy Technology 2, (2014) 921-927. http://doi.org/10.1002/ente.201402076

[3]. B. Tang, S. Huang, Y. Fang, J. Hu, C. Malonzo, D. G. Truhlar, A. Stein, Mechanism of electrochemical lithiation of a metal-organic framework without redox-active nodes, J Chem Phys 144, (2016) 194702. http://doi.org/10.1063/1.4948706

[4]. X. Han, F. Yi, T. Sun, J. Sun, Synthesis and electrochemical performance of Li and Ni 1,4,5,8-naphthalenetetracarboxylates as anodes for Li-ion batteries, Electrochemistry Communications 25, (2012) 136-139. http://doi.org/10.1016/j.elecom.2012.09.014

[5]. Y. Lin, Q. Zhang, C. Zhao, H. Li, C. Kong, C. Shen, L. Chen, An exceptionally stable functionalized metal-organic framework for lithium storage, Chem Commun (Camb) 51, (2015) 697-699. http://doi.org/10.1039/c4cc07149b

[6]. G. Li, F. Li, H. Yang, F. Cheng, N. Xu, W. Shi, P. Cheng, Graphene oxides doped MIL-101(Cr) as anode materials for enhanced electrochemistry performance of lithium ion battery, Inorganic

Chemistry Communications 64, (2016) 63-66. http://doi.org/10.1016/j.inoche.2015.12.017

[7]. L. Hu, X. M. Lin, J. T. Mo, J. Lin, H. L. Gan, X. L. Yang, Y. P. Cai, Lead-Based Metal-Organic Framework with Stable Lithium Anodic Performance, Inorg Chem 56, (2017) 4289-4295. http://doi.org/10.1021/acs.inorgchem.6b02663

[8]. K. Saravanan, M. Nagarathinam, P. Balaya, J. J. Vittal, Lithium storage in a metal organic framework with diamondoid topology – a case study on metal formates, Journal of Materials Chemistry 20, (2010). http://doi.org/10.1039/c0jm01671c

[9]. Q. Liu, L. Yu, Y. Wang, Y. Ji, J. Horvat, M. L. Cheng, X. Jia, G. Wang, Manganese-based layered coordination polymer: synthesis, structural characterization, magnetic property, and electrochemical performance in lithium-ion batteries, Inorg Chem 52, (2013) 2817-2822. http://doi.org/10.1021/ic301579g

[10]. H. Li, Y. Su, W. Sun, Y. Wang, Carbon Nanotubes Rooted in Porous Ternary Metal Sulfide@N/S‐Doped Carbon Dodecahedron: Bimetal‐Organic‐Frameworks Derivation and Electrochemical Application for High‐Capacity and Long‐Life Lithium‐Ion Batteries, Advanced Functional Materials 26, (2016) 8345-8353. http://doi.org/10.1002/adfm.201601631

[11]. D. Zhou, J. Ni, L. Li, Self-supported multicomponent CPO-27 MOF nanoarrays as high-performance anode for lithium storage, Nano Energy 57, (2019) 711-717. http://doi.org/10.1016/j.nanoen.2019.01.010

[12]. S.-B. Xia, S.-W. Yu, L.-F. Yao, F.-S. Li, X. Li, F.-X. Cheng, X. Shen, C.-K. Sun, H. Guo, J.-J. Liu, Robust hexagonal nut-shaped titanium(IV) MOF with porous structure for ultra-high performance lithium storage, Electrochimica Acta 296, (2019) 746-754. http://doi.org/10.1016/j.electacta.2018.11.135
